# The Key Role of Astrocytes in Amyotrophic Lateral Sclerosis and Their Commitment to Glutamate Excitotoxicity

**DOI:** 10.3390/ijms242015430

**Published:** 2023-10-21

**Authors:** Francesca Provenzano, Carola Torazza, Tiziana Bonifacino, Giambattista Bonanno, Marco Milanese

**Affiliations:** 1 Department of Pharmacy (DIFAR), University of Genoa, 16148 Genova, Italy; francescaprovenzano.fp@gmail.com (F.P.); carola.torazza@unige.it (C.T.); giambattista.bonanno@unige.it (G.B.); marco.milanese@unige.it (M.M.); 2 Inter-University Center for the Promotion of the 3Rs Principles in Teaching & Research (Centro 3R), 56122 Pisa, Italy; 3 IRCCS Ospedale Policlinico San Martino, 16132 Genoa, Italy

**Keywords:** astrocytes, amyotrophic lateral sclerosis, glutamate release, glutamate excitotoxicity, neuroinflammation, oxidative stress, autophagy, energy metabolism, mitochondria dysfunction

## Abstract

In the last two decades, there has been increasing evidence supporting non-neuronal cells as active contributors to neurodegenerative disorders. Among glial cells, astrocytes play a pivotal role in driving amyotrophic lateral sclerosis (ALS) progression, leading the scientific community to focus on the “astrocytic signature” in ALS. Here, we summarized the main pathological mechanisms characterizing astrocyte contribution to MN damage and ALS progression, such as neuroinflammation, mitochondrial dysfunction, oxidative stress, energy metabolism impairment, miRNAs and extracellular vesicles contribution, autophagy dysfunction, protein misfolding, and altered neurotrophic factor release. Since glutamate excitotoxicity is one of the most relevant ALS features, we focused on the specific contribution of ALS astrocytes in this aspect, highlighting the known or potential molecular mechanisms by which astrocytes participate in increasing the extracellular glutamate level in ALS and, conversely, undergo the toxic effect of the excessive glutamate. In this scenario, astrocytes can behave as “producers” and “targets” of the high extracellular glutamate levels, going through changes that can affect themselves and, in turn, the neuronal and non-neuronal surrounding cells, thus actively impacting the ALS course. Moreover, this review aims to point out knowledge gaps that deserve further investigation.

## 1. Introduction

Amyotrophic lateral sclerosis (ALS) is a fatal neurodegenerative disease affecting motor neurons (MNs) in the motor cortex, brainstem, and spinal cord [1,2,3]. The clinical traits of ALS involve adult-onset muscle weakness and wasting. Most commonly, weakness arises distally in the limb muscles and extends to the proximal muscles. Dysarthria, dysphagia, and dysphonia are relevant symptoms in about one-third of patients. In 10–15% of cases, patients also have frontotemporal dementia [4,5]. There is a high degree of variability in ALS onset, site, and progression. In most patients, survival is 3–5 years after symptom onset, with death primarily attributed to respiratory failure [6]. The incidence is 2–3 new cases per 100,000 individuals/year, and the prevalence is about 7–9 cases per 100,000 individuals [5]. Men are more at risk of developing ALS than women [7,8].

ALS patients can be familial (fALS) or sporadic (sALS). fALS patients are based on genetic mutations, usually inherited in a Mendelian autosomal dominant manner [9]. Nowadays, more than thirty mutated genes define the familial form of ALS. The most common mutations are Chromosome 9 open reading frame 72 (*C9orf72*), copper–zinc Superoxide dismutase (*SOD1*), trans-activation response DNA-binding protein 43 (*TARDPB*) and fused in sarcoma/translated in liposarcoma (*FUS/TLS*). However, fALS cases represent approximately 10% of ALS cases, while sALS includes most of ALS patients [9,10,11]. Genetic heterogeneity suggests that multiple cellular events may contribute to the disease. They include oxidative damage, mitochondrial dysfunctions, metabolic defects, protein misfolding and aggregation, impaired axonal transport, inflammation, dysregulated RNA signaling, immunological imbalance, glutamate-mediated excitotoxicity, and insufficient growth factor signaling [12,13,14,15,16,17].

The above studies aimed to unveil the pathogenic mechanisms at the basis of ALS, focusing on MN degeneration, since they represent the neuronal cells that directly lead to ALS symptoms. However, in the last two decades, non-neuronal cells, such as astrocytes, microglia, and oligodendrocytes, have been recognized to play a pivotal role in disease onset and progression. This viewpoint change derives from numerous histological observations and transcriptomic profiling of diseased tissues that unveiled a solid non-neuronal signature in neurodegenerative diseases including ALS. Several experimental pieces of evidence highlighted the non-neuronal components of ALS and identified astrocytes, microglia, and oligodendrocytes as critical players in disease onset and progression [18,19,20,21,22,23].

In this review, we focus on astrocytes in shaping the course of ALS and the mechanisms at the basis of their activity, specifically highlighting their commitment to glutamate excitotoxicity. Although the attention on astrocytes and their function in ALS progression is intensifying, studies unveiled only a limited number of altered mechanisms, thus, making urgent the need to understand which player molecules and processes are involved. Indeed, astrocytes could be a promising target to modulate the disease because of their multiple functions in maintaining CNS homeostasis.

## 2. ALS as a Non-Cell-Autonomous Disease

ALS involves different cell types, such as neurons, astrocytes, microglia, and oligodendrocytes [23,24,25]. Since all these cells express the same mutated genes in patients, ALS can arise from a combination of damaged MNs and their glial partners rather than only from the neuronal lineage. Several animal studies supported this assumption. ALS progression slowed in mutant SOD1-expressing ALS mouse models with genetic mutations restricted to neurons [20,26,27,28]. Many studies highlighted the solid non-neuronal signature in ALS and suggested astrocytes and microglia as critical players in disease progression rather than disease onset. Conversely, data support an alteration of oligodendrocyte function at the disease pre-symptomatic and early symptomatic stages [19,20,29].

### 2.1. Astrocyte Contribution to ALS

The first evidence of astrocyte alterations in ALS has derived from animal models in which mutant SOD1 was selectively expressed or deleted in these cells [30]. Pivotal experiments in chimeric animals bearing mixtures of normal cells and cells that express a human mutant SOD1 at levels sufficient to cause fatal MN disease reported that wild-type non-neuronal cells in the SOD1^G37R^ and SOD1^G85R^ chimeras delayed disease onset and prolonged mouse survival. In accordance, transgenic animals expressing mutant SOD1 in non-neuronal cells, but not in MNs, showed histological signs of neurodegeneration caused by the accumulation of ubiquitinated epitopes absent in age-matched wild-type littermates [30]. Some years later, similar experiments used Cre-Lox recombination to delete mutant *SOD1* genes selectively in microglia or astrocytes. The reduction of mutant SOD1 in the astrocytes of SOD1^G37R^ or SOD1^G85R^ mice did not delay disease onset and early disease progression. Moreover, it significantly slowed the late disease course extending mouse survival, suggesting that astrocyte dysregulation negatively controls the status of MNs at the late stage of ALS [31]. At the same time, they exhibit a more protective phenotype at the disease onset [18,28,31]. Furthermore, transplantation of wild-type astrocyte precursors in the cervical spinal cord of SOD1^G93A^ mice slowed the disease progression and prolonged survival probability [32]. Oppositely, the transplantation of astrocyte precursors carrying the SOD1^G93A^ gene mutation promoted local degeneration in the spinal cord and caused motor dysfunction in wild-type mice [33].

Further experiments validated the role of astrocytes in ALS neurodegeneration. Nagai and colleagues demonstrated the detrimental role of ALS astrocytes through in vitro co-culture studies. Mutant SOD1 astrocytes were able to induce neurodegeneration both in ALS MNs from SOD1^G93A^, SOD1^G37R^, or SOD1^G85R^ mice, as well as in healthy MNs, supporting the hypothesis of a gain of toxic functions of astrocytes in ALS [34]. Several studies demonstrated that MN viability was strongly impaired when wild-type or mutant MNs were co-cultured in direct contact with ALS astrocytes or exposed to ALS astrocyte-conditioned medium, thus encouraging the characterization of astrocyte secretome to clarify the contribution of astrocytes to disease progression [35,36,37,38,39]. Astrocytes differentiated from human fibroblasts carrying *C9orf72* mutation-induced MN death in co-culture experiments [37,39]. *C9orf72*-astrocyte RNA sequencing showed several gene alterations, including genes involved in ionotropic glutamate receptor signaling (*GRIA1, GRIA4*), complement activation, ribosomal subunit assembly, nuclear RNA export, cell adhesion (*L1CAM, TSP1, NTN1*), synapse assembly (*BDNF, NRG1, THBS2*), cell-to-cell signaling (*GPC6*), regulation of sodium ion transport (*SLC8A1, ATP1B2, NKAIN4*), and potassium ion import (*DLG1, ATP1B2*) [39]. In addition, the mutant TDP-43 or mutant VCP expression in astrocytes increased mitochondrial and ER dysfunction and induced abnormal oxidative stress in MNs, thus causing MN death [36,38]. Overall, the current literature has revealed a remarkable astrocytic dysfunction in ALS, potentially underlying molecular mechanisms that could represent a target for therapeutic approaches [40,41,42].

### 2.2. Microglia and Oligodendrocyte Contribution to ALS

For completeness, we will briefly describe the possible role of these other non-neuronal cells in the CNS.

Although the pathological mechanisms are partially obscure, microglia have a well-established relevance in ALS. Mouse SOD1^G93A^ microglia cells reduced the immune response at pre-onset stages and exhibited an anti-inflammatory behavior, then becoming mostly pro-inflammatory during disease progression [43,44]. The importance of the inflammatory signature of microglia has been highlighted by Frakes and colleagues that demonstrated a significantly reduced gliosis and increased survival following the selective partial deletion of the inhibitor of nuclear factor kappa-B kinase subunit beta (IKKB) and, consequently, NF-κB inhibition in microglia from SOD1^G93A^ mice. Knocking down IKKB in SOD1^G93A^ microglia reduced typical pro-inflammatory molecules, such as CD68, CD86, and iNOS [45].

Microglia classification in pro-inflammatory M1 and immunoregulatory M2 is challenging, and many studies showed no significant prevalence of M1 or M2 markers during the disease time course, suggesting the existence of a more complex scenario [46,47]. Accordingly, a new nomenclature should define the microglia phenotype. This scenario is even more composite since these cells can acquire individual cellular activation patterns depending on the pathological environment to which they are exposed, leading to unique disease-associated microglia (DAM) phenotypes [48].

The transcriptomic profile in single cells of spinal cord sections from SOD1^G93A^ mice and ALS patients depicted different microglia populations, varying during disease progression [49], further pointing out the diversity of microglia in ALS.

As to oligodendrocytes, ALS patient motor cortex and spinal cord showed reduced grey matter myelin and reactive changes in NG2+ cells and SOD1^G93A^ mice exhibited degeneration of grey matter oligodendrocytes before the symptom onset [20,29]. Deleting SOD1 from oligodendrocytes in the SOD1^G37R^ mouse model, proved the contribution of oligodendrocytes to the disease, showing a significant delay of the disease onset and increased survival, with no MN degeneration at the time of death [19]. These precocious alterations suggest a loss of function of oligodendrocytes, possibly in providing lactate to MNs, supporting MN degeneration [25,50]. Among the proposed mechanisms affecting oligodendrocytes functions, we can list the formation of protein aggregates, inducing ER stress, and the robust pro-inflammatory environment characterized by a high level of interferon γ (IFN-γ) [20], and dysregulation of myelination and lipid signaling pathways [51].

A significant survival reduction in MN/SOD1^G93A^ oligodendrocytes co-cultures compared to co-cultures with control oligodendrocytes has been reported [52]. The authors obtained similar results when co-culturing MNs with oligodendrocytes being differentiated from sporadic, and mutated SOD1, C9orf72, or TDP-43 fibroblasts generally reducing MN survival based on the decreased lactate production and transport in the oligodendrocytes, leading to an energetic deficit in the MNs [52]. Ferraiuolo and colleagues silenced SOD1 in mutated human and mouse oligodendrocytes and observed an increase in MN survival, indicating a SOD1-dependent toxic mechanism.

As for the lactate support to MNs by oligodendrocytes, a first work demonstrated a reduced expression of monocarboxylate transporters (MCTs) in the motor cortex of ALS patients [25] and rodent and canine models of the disease [53,54]. In mouse SOD1 mutants, *MCT1* transcripts were downregulated in early and late-symptomatic mouse spinal cord ventral horn grey matter [53]. Overexpressing misfolded SOD1 in zebrafish mature oligodendrocytes also induced disruption of the myelin sheaths and MCT-1 downregulation [55] along with behavioral abnormalities, such as thigmotaxis, freezing behavior, erratic movements, and learning impairment [56].

## 3. Astrocyte Mechanisms Fostering Neuronal Damage in ALS

Astrocytes are the primary cell type regulating homeostasis in the CNS and are very specialized and heterogeneous throughout the CNS. They control extracellular ion concentration, maintain blood–brain barrier integrity, promote myelination in the white matter, and support neurons [57]. Astrocytes play a fundamental role in synapse regulation. Peri-synaptic astrocytes abundantly express various transporters that maintain neurotransmitter homeostasis in the synaptic cleft. A high plastic capacity characterizes these astrocytes, which participate in synaptogenesis, synaptic maturation, and synaptic extinction [58]. Apart from the fundamental astrocyte physiological functions, their dysfunction can generate neurological disorders such as neurodegenerative or neurodevelopmental diseases, epilepsy, and astrogliomas [59].

In response to a damaging insult, astrocytes shift from rest to a highly reactive and proliferative phenotype with supportive characteristics to mend the damage by supplying trophic factors and reducing neuronal degeneration. In many neurodegenerative diseases, this mechanism is impaired and leads to neurotoxic events [60]. Indeed, the influence of astrocytes is more complex and can be beneficial or detrimental depending on the disease and the pathological conditions [61]. Accordingly, distinctive molecular and functional profiles characterize the reactive astrocytes and their impact on diseases and produce unique astrocyte phenotypes [62]. Many studies have suggested that astrocytes can act as two distinct reactive categories, the A1 neurotoxic phenotype and the A2 neuroprotective one [63,64,65]. However, scientists should consider this dual polarization with caution since more recent studies proposed moving beyond the A1–A2 classification since only a subset of transcripts related to the A1 and A2 states are upregulated in patient or mouse models of CNS disease astrocytes, and multidimensional data support the idea that A1 and A2 are just two of many potential transcriptomes of astrocytes [66,67,68,69].

Thus, astrocytes may play a fundamental role in shaping CNS disease genesis and progression. In the following sections, we will describe the impact of astrocytes on the main etiological mechanisms involved in ALS.

**Figure 1:**
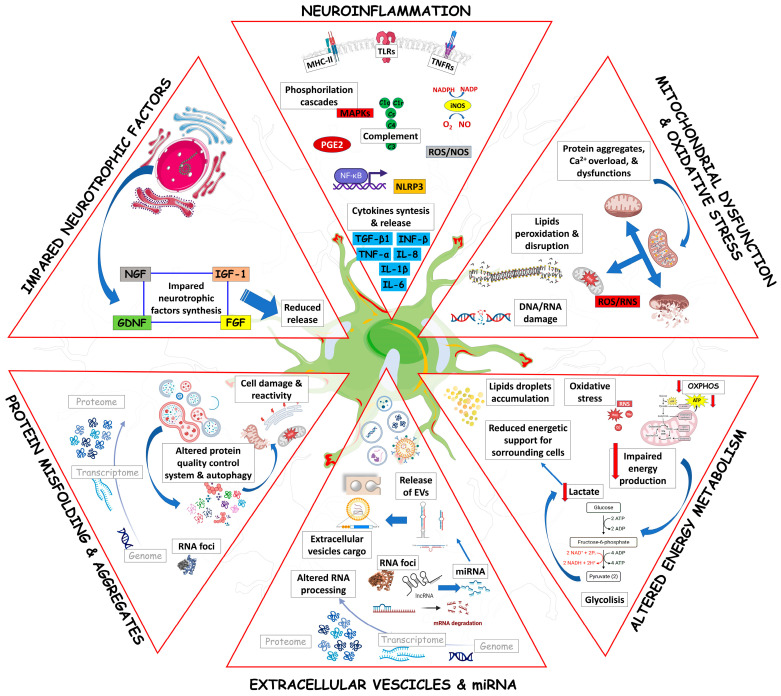
The mechanisms that characterize ALS astrocytes fostering neuronal and glial damage are schematically represented. Neuroinflammation, mitochondrial dysfunction, oxidative stress, energy metabolism impairment, miRNAs and extracellular vesicle involvement, protein misfolding, autophagy dysfunction, and neurotrophic factor dysregulation are major phenomena in ALS progression. The contribution of glutamate and excitotoxicity has been reported in detail in Figures 2 and 3. The figure was generated using BioRender.com (accessed on 25 July 2023) and Servier Medical Art, provided by Servier, licensed under a Creative Commons Attribution 3.0 unported license (https://creativecommons.org/licenses/by/3.0/ (accessed on 25 July 2023)).

Figure 1 schematically summarizes the main pathogenic mechanisms that affect ALS astrocytes, concurring with their aberrant activation and neurotoxicity.

### 3.1. Astrocytes and Neuroinflammation in ALS

Among the several functions of astrocytes, one is the regulation of innate immunity in the CNS. Astrocytes express numerous receptors and produce factors involved in immunological response activation, such as Toll-like receptors, inducible nitric oxide synthase (iNOS), major histocompatibility complex-II, nuclear factor kappa-light-chain-enhancer of activated B cells (NFkB) and mitogen-activated protein kinases (MAPKs) [70,71,72]. The evidence that astrocytes actively modulate the inflammatory response and the increased serum/plasma and cerebrospinal fluid (CSF) levels of tumor necrosis factor-α (TNF-α), interleukin-6 (IL-6), interleukin-8 (IL-8), and interferon-β (INF-β) [73] encouraged the study of astrocytes as inflammatory mediators in ALS.

A recent analysis described astrocyte alteration following transient ischemia induced by the rat treatment with the bacterial endotoxin lipopolysaccharide (LPS) to generate an inflammatory response in the brain cortex or by the middle cerebral artery occlusion (MCAO) to produce an ischemic status. LPS-treated astrocytes showed an increased transcription of the genes related to classical complement cascade activation, such as complement components C1r, C1s, C1q, C3, and C4 that play a critical role in synapse pruning during development and likely lead to synapse loss in neurodegenerative diseases. On the other hand, after MCAO, most of the upregulated genes were associated with the production of neurotrophic factors and cytokines, including corticotrophin-like cytokine factor 1 (CLCF1), leukemia inhibitory factor (lif), and IL-6, IL-10 and thrombospondins that facilitate the regeneration of lost synapses. These different transcriptional patterns demonstrate that astrocyte reactive gliosis is a highly heterogeneous state, which differently alters astrocytes to respond to the specific disease evolution stage [63], thus raising the question of how many subtypes of reactive astrocytes exist beyond the simplified A1 and A2 classification mode.

The following study indicated that LPS-stimulated M1-like microglia induced A1-like astrocyte toxic activation by secreting interleukine-1α, IL-1β, TNF-α, and C1q [64]. In ALS and most neurodegenerative diseases, neuroinflammation and M1-like activated microglia were described, suggesting its activation of A1-like astrocytes. Indeed, C3, highly upregulated in A1-like and absent in A2-like astrocytes, was massively represented in post-mortem tissues from ALS, Alzheimer’s disease, Huntington’s disease, Parkinson’s disease, and multiple sclerosis. This evidence demonstrates that A1-like astrocytes are present in most neurodegenerative diseases and probably retain a non-marginal role in neuronal death [64,74].

It is becoming clear that astrocytes in neurodegenerative diseases, including ALS, are subjected to a shift from a supportive to a neurotoxic phenotype, causing the metabolic alteration, loss of trophic function, secretion of toxic factors, and development of a chronic inflammatory response [60]. In ALS, this assumption founded the basis of the pivotal reports showing that down-regulation of SOD1^G37R^ or SOD1^G85R^ in astrocytes of transgenic mice did not affect the early phases of the disease but ameliorate clinical symptoms of the late disease course and survival [18,31,75]. Recent studies confirm the different astrocyte signatures and activation states cultured from neonatal and adult SOD1^G93A^ mice. Indeed, primary astrocyte cultures from the neonatal SOD1^G93A^ mouse brain did not show upregulation of the classical pro-reactive astrocyte genes, such as inflammatory genes and the reactive factor lipocalin 2 (Lcn2). On the contrary, astrocytes prepared from 2-month-old or late symptomatic stage SOD1^G93A^ mice recapitulated the typical phenotype observed in post-mortem astrocytes from ALS patients [76,77,78]. This evidence also highlights the importance of in vitro studies focusing on the in vivo maturation of astrocytes during disease progression, spanning from the pre-symptomatic/low-progressing to the symptomatic/fast-progressing stages, which determine their activation upon in vivo exposure to an authentic pathological environment.

ALS patients are characterized by high transforming growth factor-β1 (TGF-β1) levels in serum, plasma and CSF [79]. Abnormal expression of TGF-β1 has also been detected in astrocytes from sporadic ALS patients and SOD1^G93A^ mice, causing a faster disease progression. This neurotoxic effect seems to derive from a TGF-β1-dependent alteration of the balance between interferon-γ (INF-γ) and interleukin-4 (IL-4) production in T cells and microglia. The pharmacological treatment after the disease onset with a TGF-β signaling inhibitor prolonged the survival of SOD1^G93A^ mice, rescuing INF-γ/IL-4 dysregulation and decreasing the number of activated microglial cells. Moreover, the correlation between TGF-β1 level and the disease rank suggested using TGF-β1 as a predictive biomarker for disease progression and severity [80]. In addition, increased TGF-β1 secretion by astrocytes was reported to activate the mammalian target of the rapamycin (mTOR) signaling pathway, inducing aggregation of sequestosome-1 and microtubule-associated protein 1A/1B-light chain 3-II and leading to an impairment of autophagy in MNs [76].

TNF-α is one main cytokine overexpressed in the blood and CSF of ALS patients [81,82,83]. High TNF-α concentrations and TNF-α receptor (TNFR) up-regulation have also been reported in the spinal cord of SOD1^G93A^ mice before the symptom onset [84,85]. TNF-α can be differentiated into membrane-bound TNF-α (mTNF-α) and soluble TNF-α, mediating cytotoxic and apoptotic effects through TNFR1 and TNFR2 activation. TNRF2 can induce tumoral and neuronal cell death by binding mTNF-α. This mechanism seems strongly involved in ALS. Indeed, a study in spinal cord astrocytes/MN co-cultures from SOD1^G93A^ mice reported the mTNF-α increase in MNs and the reduction of TNRF2, not of TNRF1, ultimately rescued MNs. The same MN survival-linked positive effects were observed in TNFR2 knocked-out SOD1^G93A^ mice [86]. Oppositely, TNRF1 ablation exacerbated the detrimental effects of TNF-α by decreasing the production and secretion of the glial-derived neurotrophic factor from astrocytes [85]. Another study demonstrated that both receptors exert their functions by activating the apoptosis signal-regulated kinase 1 (ASK1)/p38MAPK pathway, which was harmful to MNs. p38MAPK inhibition prevented MN death in SOD1^G93A^ mouse-derived astrocyte/MN co-cultures, suggesting the essential role of the TNFR/ASK1/p38MAPK pathway in neurodegeneration [87]. TNF-α exerts its toxic function also in other models of ALS. For instance, mutant FUS-expressing astrocytes secreted TNF-α as the primary toxic factor mediating MN death through the NFκB pathway activation. Moreover, TNF-α modulated the expression of α-amino-3-hydroxy-5-methyl-4-isoxazolepropionic acid (AMPA) receptors and GluA2 AMPA subunit in MNs, determining an increased permeability to Ca^2+^ and leading to excitotoxicity [88]. In addition, FUS-overexpressing astrocytes produce a robust inflammatory response by abnormal TNF-α, IL-1β, and IL-6 secretion and augmented transcription of inducible nitric oxide synthase (iNOS) and prostaglandin E2 (PGE2) [89]. The cytosolic nucleotide-binding oligomerization domain-like receptor pyrin domain containing 3 (NLRP3) is one principal mediator of neuroinflammation in ALS, in other neurodegenerative diseases, and during brain aging [90,91,92]. The SOD1^G93A^ rat brain possesses increased NLRP3 and active caspase 1 levels, and this augmentation was associated with a higher NFκB expression [93]. Johann and colleagues identified astrocytes as the primary cell population expressing NLRP3 in ALS. They detected excessive NLRP3 and IL-1β concentrations, co-localized with the glial fibrillary acidic protein (GFAP) in the spinal cord of 60 days old SOD1^G93A^ mice and post-mortem ALS patient tissue [94]. A subsequent paper hypothesized that one cause for the increased NLRP3 expression is protein nitration due to reactive oxygen (ROS) and nitrogen (RNS) species, highly produced and secreted in ALS. The treatment of SOD1^G93A^ microglia with iNOS and NAPHH oxidase 2 inhibitors reduced the nitrotyrosine levels and, consequently, caspase 1 and NLRP3 activation, supporting the involvement of protein nitration in the neuroinflammation spread [95].

Finally, a link between neuroinflammation and S100β, a Ca^2+^ binding protein expressed selectively in astrocytes, was described. S100β behaves as a component of the danger-associated molecular pattern signaling and, when released in high concentrations, participates in the cascade of events causing cell injury and binds the receptor for advanced glycation end products (RAGE), leading to microglia migration. S100β silencing in SOD1^G93A^ astrocytes determined the down-regulation of GFAP, TNF-α, C-X-C motif chemokine 10 and Chemokine (C-C motif) ligand 6 expression, ameliorating the reactive pro-inflammatory phenotype of ALS astrocytes [96].

The above evidence highlights the essential role of astrocyte in supporting the inflammatory response. However, further studies are required to clarify the dual function of astrocyte-released cytokines during ALS progression and to understand the timeline of astrocyte phenotypes shifting to pro-inflammatory ones.

### 3.2. Astrocytes, Mitochondrial Dysfunction, and Oxidative Stress in ALS

Oxidative stress is one of the best-studied subjects in ALS research. Oxidative stress biomarkers are present in ALS patients’ urine, blood, CSF, and individual tissues [97,98]. Oxidative stress derives from the imbalance between oxidants and antioxidants within a biological structure. Abnormal production of ROS or a deficit in antioxidant systems might be the basis of tissue damage and cell death [99]. Hydrogen peroxide (H_2_O_2_), superoxide anion (O_2_^•−^), and hydroxyl radical (HO^•^), but also (RNS), such as nitric oxide (NO), are physiologically produced during the cell life cycle. Indeed, many cell functions, such as signal transduction, gene transcription, oxidative phosphorylation, and ATP production in mitochondria, require oxygen as a substrate and generate H_2_O_2_, O_2_^•−^, or HO^•^ through redox reactions [100]. We know that, under massive oxidative stress, when antioxidant enzymes, such as glutathione peroxidase, SOD1, and catalase, do not detoxify the cells, the accumulation of reactive molecules, damage the cell structure by causing the oxidation of different biomolecules, such as lipids, protein, and DNA/RNA [101].

The mitochondrion is the cell compartment where most ROS/RNS originate, probably because mitochondria are enriched in redox enzymes [102]. Mitochondria dysfunctions, e.g., due to mitochondria DNA damage, could improve ROS/RNS production and reduce protective action from these reactive species. Since mitochondria are the principal producers of the energy needed by cells, their malfunction leads the cells to apoptosis or senescence, producing catastrophic events, especially in non-proliferative cells, such as neurons [103,104]. In addition, a mitochondrial deficit can cause ER stress, exacerbating the dysregulation of Ca^2+^ homeostasis and promoting abnormal protein folding and aggregation [105]. Mitochondrial and ER dysfunctions also determined communication impairment between these two organelles [106]. For instance, defects in mitochondria-associated ER membranes (MAMs) in iPSC-derived FUS MNs showed detrimental effects on axonal transport and ATP availability for neuronal survival, further emphasizing the importance of MAMs in cell homeostasis [107].

Astrocytes help neurons to prevent and counteract oxidative stress damage in physiological conditions by releasing glutathione, antioxidant enzymes, and ROS/RNS scavengers [108]. On the other hand, primary astrocytes prepared from SOD1^G93A^ rats exacerbated oxidative stress through the abnormal NO production and secretion, sensitizing wild-type MNs to nerve growth factor (NGF)-induced apoptosis through p75 neurotrophin receptor (p75NTR) signaling. The transcription factor nuclear factor erythroid 2-related factor 2 (Nrf2) induction in SOD1^G93A^ astrocytes, which decreases in ALS patients [109], rescued MN degeneration by promoting glutathione biosynthesis, which abolished NGF/p75NTR-induced apoptosis [110]. In accordance, the selective Nrf2 over-expression in SOD1^G93A^ mouse astrocytes determined delay of disease onset, lifespan extension, and gliosis decrease [111]. A very recent study also demonstrated that specific miRNAs shuttled by extracellular vesicles (EVs) from mesenchymal stem cells (MSCs) can induce the nuclear translocation of Nrf2 and the expression of the antioxidant factor NQO1, reducing the accumulation of oxidative species and, in turn, the reactive phenotype and neurotoxicity of mouse- and human-derived ALS astrocytes [77]. Another study emphasized the Nrf2 involvement in protecting neurons from oxidative stress [112]. In response to oxidative stimuli, neurons release angiogenin (ANG), which binds the syndecan-4 receptor and activates the protein kinase Cα (PKCα). In turn, PKCα phosphorylates Nrf2, upregulating its transcriptional activity. To confirm the ANG and Nrf2 neuroprotective characteristics, primary neonatal astrocytes from antioxidant-responsive element-human placental alkaline phosphatase (ARE-hPAP) transgenic mice or Nrf2 knock-out mice were treated with ANG. ARE–hPAP astrocyte-conditioned medium supported cell survival when applied to neurons exposed to H_2_O_2_. The Nrf2 knock-out mouse astrocyte-conditioned medium had no neuroprotective effects [112]. ANG gene mutations are associated with ALS disease, leading to loss of function [113,114]. Indeed, the astrocyte ARE–hPAP stimulation with the ANGH114R mutated variant did not benefit cell survival [112].

Because of the key role of Nrf2 in regulating antioxidant response, many studies focused on administering Nrf2 activators to slow disease progression. Two analogues of the 2-cyano-3,12-dioxooleana-1,9-dien-28-oic acid were tested in the SOD1^G93A^ mouse model. Nrf2 function was recovered in treated mice, and the upregulation of classical Nrf2-regulated genes was registered. These positive biomolecular effects improved motor performance, reduced weight loss, and prolonged survival [115]. Then, other Nrf2 activators were tested, such as tert-butylhydroquinone, DL-sulphoraphane, lipoic acid, fumaric acid and curcumin [109]. The unsuccessful results of these positive modulators have been ascribed to their low blood-brain barrier penetration. However, they could also relate to the still undefined mechanism of action. Interestingly, the expression of a large variety of non-Nrf2-dependent genes was modified from these substances [116].

Along with the oxidative stress defense impairment, increased production of ROS and NOS is present in ALS due to mitochondrial dysfunction [117,118,119]. Cassina and colleagues reported that rat spinal cord SOD1^G93A^ astrocytes exhibit defective mitochondrial respiration. Indeed, stimulation of the mitochondrial activity in the presence of ADP did not enhance oxygen consumption and ATP synthesis in SOD1^G93A^ astrocytes compared to controls. One of the possible causes of mitochondria malfunction refers to nitroxidative stress, confirmed by the observation that the treatment with antioxidants and the inhibition of NOS generation partially restored the mitochondrial respiratory chain function [120]. A following study carried out on gliosomes, an ex vivo model of the peri-synaptic astrocyte processes, isolated from the spinal cord of 30, 60, 90, and 120 days old SOD1^G93A^ mice, described a substantial increase in lipid peroxidation already at 30 days of age. However, dysfunction of mitochondrial respiration and, therefore, reduction of ATP/AMP ratio was recorded only at 90 and 120 days, representing symptomatic stages of the disease [119]. These data support the idea that elevated oxidative stress is one of the first events occurring in ALS astrocytes, leading to toxicity in the neuronal compartment.

In addition, primary astrocytes isolated from transgenic mice carrying ALS-causing SOD1 mutations (SOD1^G93A^ or SOD1^G86R^) or TDP-43 (TDP-43^A315T^) induced ROS/NOS in MNs, due to the astrocytic release of toxic factors. Astrocyte-conditioned medium induced MN death by activation of Nav channels and nitroxidative stress. Treating MNs with Nav channel blockers, such as mexiletine, spermidine, or riluzole, before exposure to the astrocyte-conditioned medium abolished ROS/NOS formation and prevented MN death [36]. The ALS astrocyte-mediated opening of the Nav channel in MNs might be one of the first events in the detrimental cascade leading to neuronal loss. Indeed, Na^+^ influx, induced by mutant SOD1 and TDP-43 astrocyte-conditioned medium, caused membrane depolarization and, consequently, Ca^2+^ entrance in MNs. To counteract the massive increase in Ca^2+^, mitochondrion Ca^2+^ content was overloaded, causing mitochondria damage and ROS/NOS generation. Lastly, high levels of ROS/NOS promote the phosphorylation and activation of Abelson murine leukaemia viral oncogene homolog 1 (c-Abl) signaling pathway and MN death, which was prevented by the c-Abl inhibitor STI571 or by antioxidants [118].

Targeting oxidative stress and mitochondrial dysfunction has always been a promising approach for treating ALS [121]. Therefore, many drugs counteracting oxidative stress and ameliorating mitochondria response have been tested during the last decades [122]. Among these, dichloroacetate inhibits pyruvate dehydrogenase (PDH) kinase (PDK), maintaining PDH in the unphosphorylated active form and increasing coenzyme-A formation from pyruvate. Treatment with dichloroacetate promoted the mitochondrial respiration rate and regulated the proliferation of rat SOD1^G93A^ astrocytes, which positively affected MN survival in co-culture experiments [123]. Moreover, in vivo studies administering dichloroacetate to SOD1^G93A^ rats delayed the disease onset, preserved the neuromuscular junctions, normalized gliosis, and prolonged survival probability [124]. These promising results should encourage us to keep studying mitochondria alterations in the different CNS cell types to identify specific pathways at the basis of pathological dysfunction and to develop cell-targeted therapies. Of note, edaravone, one of the two drugs approved for ALS treatment, is a free-radical scavenger [125].

### 3.3. Astrocytes and Energy Metabolism in ALS

Astrocytes represent one primary energy source for MNs, mainly through the shuttle of lactate [126]. MNs can promote aerobic glycolysis in astrocytes by glutamate release. This latter induces the activation of the Na^+^/K^+^-ATPase pump that consumes the ATP produced by phosphoglycerate kinase (Pgk), triggering glucose uptake and glycolytic processing, leading to the release of lactate from astrocytes. Lactate can then contribute to the activity-dependent fueling of the neuronal energy demands associated with synaptic transmission [127]. However, gene expression analysis in SOD1^G93A^ astrocytes revealed an impairment of many proteins involved in the lactate shuttle, such as GLAST-1, Na^+^/K^+^ -ATPase, Pgk and the lactate efflux transporter Solute carrier 16a4 (Slc16a4).

The quantification of lactate levels in the spinal cord of SOD1^G93A^ transgenic mice validated the gene analysis results. SOD1^G93A^ mice showed a significant reduction of lactate shuttle between astrocytes and MNs at an early stage, followed by a further decrease during disease progression. SOD1^G93A^-derived astrocytes showed the same impairment of lactate shuttle when co-cultured with transgenic or wild-type mouse MNs [128,129]. Moreover, the lactate transporter Slc16a4 expression was downregulated in spinal cord iNPC-derived astrocytes from three patients with ALS carrying SOD1 mutations compared to control individuals. Impairment of the lactate shuttle led to MN energy deficit, causing membrane potential alteration and ion concentration imbalance [128].

Metabolic analysis of culture medium collected from SOD1^G93A^ mouse-derived astrocytes pointed out an altered composition of metabolites, highlighting a dysregulation of arginine, proline, lysine, glutathione, glycerophospholipid, and glycolysis or gluconeogenesis metabolism markers. This evidence supports a significant dysregulation of excitotoxicity, mitochondrial dysfunction, and oxidative stress pathways in MNs and astrocytes [129,130].

Metabolism dysregulation also occurred in gliosomes isolated from the spinal cord and motor cortex of SOD1^G93A^ mice that showed increased glycolysis and lactate fermentation at the symptomatic but not at a pre-symptomatic stage of the disease. However, these alterations occurred at more precocious stages of the disease in the neuronal counterpart, indicating precocious changes at the presynaptic level and later in the peri-synaptic region. Interestingly, the augmented lactate dehydrogenase activity in gliosomes could be the astrocyte response to the impairment of the lactate shuttle to attempt supplying more energy to MNs [131]. In addition, rat astrocytes facilitated anaerobic glycolysis and increased lactate production when transfected with mutant TDP-43208-414. Although the lactate concentration was high in TDP-43208-414-transfected astrocytes, its transfer was impaired due to the astrocyte-specific monocarboxylate transporter-1 (MCT-1) downregulation [132].

The metabolism of nucleosides was also altered in astrocytes from *C9orf72* and sporadic ALS patients. ALS models showed an increased release of ATP from MNs, determining microglia activation and increased astrogliosis and neuroinflammation. Moreover, adenosine, the last step of ATP metabolism, was found to be significantly elevated in the cerebrospinal fluid of ALS patients [133]; concomitantly, the expression of adenosine A2A receptors (A2ARs) is also increased in the spinal cord of ALS patients and SOD1^G93A^ mice [134,135]. Acting on A2AR, adenosine promotes astrocyte proliferation and activation, reduces glutamate uptake, and stimulates Ca^2+^-dependent glutamate release [136,137]. The up-regulated adenosine levels were related to a lower adenosine deaminase (ADA) expression in ALS astrocytes, which converts adenosine into inosine. As a proof-of-concept, the inhibition of ADA in control astrocytes caused an impairment of MN viability in astrocyte/MN co-cultures. In addition, exposure of ALS astrocytes to inosine partially rescued MN survival, validating the relevant function of adenosine and inosine in ALS [138].

Recently, a study on energy metabolism revealed that astrocytes from *C9orf72* patients were subject to a generally reduced metabolic flexibility, increasing their sensitivity to starvation-induced stress. Indeed, *C9orf72* repeat expansion altered the transport of energy substrates and caused defects in fructose and glycogen metabolism, thus promoting the formation of advanced glycation end products and the glycogen accumulation in the CNS due to its mobilization impairment following high-energy demand. The picture highlights the deprivation of mitochondrial energy substrate availability and a propensity to shift toward a more glycolytic ATP production state in the C9orf72 astrocytes [139].

It is well-established that ALS patients exhibit lipid hypermetabolism [140,141]. Lipidomic analysis of the spinal cord from SOD1^G93A^ rats showed cholesteryl esters and cardiolipin augmentation, and ceramide metabolism alteration [142]. Interestingly, astrocytes derived from SOD1^G93A^ rats or rat astrocytes transfected with mutant TDP-43208-414 presented an accumulation of lipid droplets (LDs) [132,143]. The impact of LD accumulation in glial cells is still controversial. Indeed, reduced LD deposition in glial cells slowed neurodegenerative processes in Drosophila mutants, affecting mitochondrial function [144]. However, a subsequent study demonstrated that ROS could induce abnormal lipid synthesis in neurons. Because high levels of lipids can promote lipid peroxidation and cellular damage, neurons transfer lipids to glial cells as a protective mechanism [145].

### 3.4. Astrocytes, miRNAs and Extracellular Vesicles in ALS

The miRNA expression levels are modified in ALS animal models. Cortical astrocytes from SOD1^G93A^ mice showed a down-regulation of miR-146a, miR-125 and miR-21 compared to control astrocytes [146]. miR-146a works as a negative feedback regulator of the TLR inflammatory pathway [147]; therefore, its downregulation, already observed at the early stages of the disease, could be one of the causes of the augmented HMGB1 and NFκB levels in SOD1^G93A^ astrocytes. MiR-125 and miR-21 are modulators of neurite outgrowth [148,149], thus making them potential toxic factors involved in impairing the synaptic structure and function [146].

At the same time, the pattern of miRNAs released from astrocytes through extracellular vesicles (EVs) is also altered. MiRNAs secreted by astrocytes regulate several transcripts encoding proteins involved in axonal growth and maintenance. EV cargo was modified in *C9orf72* astrocytes and was able to induce MN toxicity. MiR-494-3p was strongly down-regulated in EVs derived from *C9orf72* human astrocytes, determining impairment of axonal development, and causing neurite retraction and MN death by increasing Semaphorin-3A levels [150]. The study of the altered miRNA pattern represents a growing field in ALS. Identifying circulating miRNA modification, particularly at pre-symptomatic and early stages of the disease, can generate a panel of biomarkers to diagnose ALS precociously. Saucier and colleagues described a miRNA signature in EVs from ALS patient plasma, identifying a variety of miRNAs involved in the regulation of known ALS-linked altered functions, such as the Nrf2 pathway, Wnt/β-catenin axis, transcription, and protein ubiquitination [151]. Another study focused on neuron-derived EVs in ALS patients’ plasma identified a panel of modified miRNAs related to synaptic vesicle docking and exocytosis, regulation of neurotransmitter secretion, and synaptic vesicle cycle [152]. These are only two examples of a broader and promising landscape that needs a systematic analysis for functional and translational readouts.

EVs may be a way to eliminate toxic substances from the affected cells and may represent a mode for disease spreading, including in ALS. Mutant and misfolded wild-type SOD1 can propagate from different cells to recipient cells in association with the vesicles released into the extracellular environment [153], and it has been shown that SOD1^G93A^ mouse exosomes derived from primary astrocytes contain and sprout abnormal amount of mutant SOD1, provoking MN death [154,155]. Similar findings come from other cell types. For instance, the exposure of Neuro2a cells to exosomes from the brains of TDP-43^A315T^ mice, but not from the control brains, caused cytoplasmic redistribution of TDP-43, suggesting that secreted exosomes contributed to the propagation of the TDP-43 proteinopathies. However, blocking EV production in vivo exacerbated the disease progression of mice expressing human TDP-43^A315T^. The in vivo data suggest that EV secretion is overall beneficial in the neuronal clearance of pathological TDP-43 [156]. Increased SOD1, TDP-43, and FUS levels were reported in EVs from the plasma of sporadic ALS patients [157]. EVs-containing wild-type or mutant (R521G or R495X) FUS were also isolated from SH-SY5Y and N2A cells and showed higher mutant FUS levels than wild-type FUS [158]. Finally, primary rat cortical neurons, transfected with plasmids encoding poly(GA)50, poly(GP)50, poly(GR)50, poly(PA)50, and poly(PR)50, and FLAG-GFP, and iPSC-derived human neurons carrying the *C9orf72* mutation were able to transfer dipeptide repeat (DPR) proteins both through cell-to-cell contact and the EV release, thus spreading DPR aggregate toxicity to primary control astrocytes [159].

Despite the evidence supporting the astrocyte secretome toxicity in ALS, the content of EVs released from astrocytes is only partially uncovered. Understanding cell-to-cell communication mechanisms is the leading step to evaluate CNS functioning. Because of the large variety of CNS functions and the essential role of EVs in cell-to-cell communication, more efforts to comprehend the astrocyte EV release regulation and the cargo composition and their texting with neurons and microglia could also drive the identification of new pathogenic mechanisms in ALS.

### 3.5. Astrocytes and Protein Misfolding and Autophagy in ALS

Autophagy impairment represents a well-established ALS feature in MNs and glial cells [160,161,162]. Many ALS genes are autophagy regulators, such as *OPTN*, *SQSTM-1* and *TBK-1*; in addition, proteins encoded by the genes *C9orf72*, *VCP*, *CHMP2B*, *VAPB*, *ALS2*, and *DCTN1* modulate vesicular trafficking and autophagosome assembly; therefore, they may affect autophagy [163]. Given the crucial role of autophagy in balancing the beneficial and detrimental effects of immunity, inflammation, and metabolism in other cell types [164], autophagy appears an attractive target for the titration of cell activity to ultimately slow down disease progression. Most studies focused on autophagy contribution in the MNs, and the involvement of autophagy in glial cell dysfunction still needs to be included. Despite the progress that has been made in understanding the basic mechanistic principles underlying autophagy, many unanswered questions remain for cell type-specific roles of autophagy and autophagy-related pathways, as well as regarding the contribution of defects in these processes to the onset and/or progression of neurodegenerative diseases. Indeed, the non-cell autonomous mechanisms leading to autophagy impairment are still undefined.

Little is known about the role of autophagy in astrocytes. Astrocytes actuate the phagocytic and secretive processes linked to the activity of inflammatory molecules, such as IL-1β and IL-18. Like microglia, these cell types might also share the involvement of autophagy in immune-related processes [165,166]. However, evidence for this hypothesis is lacking.

A link between glutamate processing by astrocytes and autophagy has been proposed [167]. Increased cytosolic Ca^2+^ concentration led by glutamate uptake into astrocytes may activate the autophagic pathways. While Ca^2+^ elevations can trigger gliotransmitter release, autophagy induction could provide metabolic support for astrocytes and nearby neurons. Furthermore, parts of the autophagy machinery could play a role in releasing molecules from astrocytes, as described for the insulin-degrading enzyme in murine primary astrocytes [168,169]. The finding that the ATP release into the extracellular space involves autophagic vesicles in HeLa cells, melanoma cells, and rat primary astrocytes supports this notion [170,171,172,173]. However, the exact role of autophagy in astrocyte unconventional secretion remains to be established.

Interest in the astrocyte autophagy role in ALS is increasing since astrocyte contribution to ALS has been largely substantiated. In a mutant SOD1 mouse ALS model, inclusions appear in astrocytes first and to a greater extent than in neurons [174]. Accordingly, astrocyte-specific attenuation of mutant SOD1 expression slowed disease progression in transgenic mice [18,175]. Pro-inflammatory conditions in neurodegenerative diseases can induce profound changes in the astrocyte mitochondrial network. Under these conditions, fragmentation of mitochondria and decreased respiratory capacity occur [176]. Therefore, functional autophagy is indispensable for astrocytes in an inflammatory environment to maintain the mitochondrial architecture and prevent ROS accumulation. In ALS mice models with mutated FIG4 and VAC14, most p62-containing inclusions were in astrocytes, highlighting the relevance of autophagic clearance in these cells [177].

Moreover, astrocytes from mice with autophagic-lysosomal dysfunction contribute directly to neurodegeneration due to an impaired ability to metabolically support neurons [178]. Astrocytes may also assist neurons in the degradation of their waste. Neurons extrude protein aggregates and damaged organelles, which are subsequently taken up and degraded by *C. elegans* and murine astrocytes [179,180].

In this context, natural polyamines (putrescine, spermidine and spermine) are ubiquitous molecules known to regulate several physiological processes, including triggering autophagy, and result altered in the spinal cord of ALS mouse model, especially in the spinal cord and at the late stage of the disease [181]. Moreover, polyamine system dysregulation has also been found in human ALS patients [182]. Polyamines can be released from and stored in astrocytes [183,184,185]. In the first case, these molecules regulate the function of receptors and channels in glia and neurons [183], while in the second condition, they affect their own glial functions [186,187,188]. How polyamines affect autophagy in ALS astrocytes has yet to be elucidated and deserves better investigation since it could be a promising target for modifying disease progression.

The notion that several astrocyte-supportive roles involve the autophagy pathway highlights the importance of assessing the impact of ALS-causing mutations in autophagy components on astrocyte function in ALS.

### 3.6. Astrocytes and Neurotrophic Factors in ALS

Astrocytes secrete many trophic factors, including nerve growth factor (NGF), glial cell line-derived neurotrophic factor (GDNF), insulin growing factor-1, and fibroblast growing factors (FGF) [189], especially during ALS [190], mature nerve growth factor (NGF) modulates neuronal differentiation and survival by binding to the Tyrosine receptor Kinase A (TrKA). On the other hand, Pro-NGF preferentially binds the p75 receptor, which determines axonal growth and remodelling during development, although its expression is barely detectable in adults. Notably, p75 expression increases in pathological conditions, promoting the activation of apoptotic pathways by boosting NFκB, p53 and Bax. Ferraiuolo and colleagues demonstrated that astrocytes derived from SOD1^G93A^ mice expressed a higher amount of Pro-NGF compared to WT astrocytes and, at the same time, the p75 receptor and its pro-apoptotic associate protein were upregulated in SOD1^G93A^ astrocytes. This alteration and a reduction of mature NGF decrease MN viability in ALS mice [191]. GDNF is considered a strong protective factor promoting neuronal survival, and many efforts have been made to translate this evidence into an effective ALS therapy [192]. A recent study tested the systemic injection of AAV9-GDNF in SOD1^G93A^ rats. However, these experiments registered only a modest functional improvement of motor performance and any effects on survival in SOD1^G93A^ rats.

Moreover, AAV9-GDNF administration caused side effects, including slowed weight gain, reduced overall activity levels and impaired working memory [193]. Interestingly, a subsequent study showed that hNPCs expressing GDNF that differentiate in vivo into astrocytes ameliorated the health of the upper MNs, supported lower MN survival, delayed paralysis, and extended lifespan when transplanted in the cortex of SOD1^G93A^ rats. The FDA has approved these cells for clinical trials to explore their safety and efficacy in ALS patients (ClinicalTrials.gov Identifier: NCT02943850) [194].

### 3.7. Astrocytes and Glutamate Excitotoxicity in ALS

Glutamate is the most engaged neurotransmitter in the mammalian CNS, mediating excitatory neurotransmission. However, excessive glutamatergic input elicits excitotoxicity in ALS [195,196,197,198,199,200,201], starting from the pre-symptomatic phases of the disease and contributing to neurodegeneration.

Excessive glutamate can originate from neuronal release, and also indirectly from a reduced uptake. Synaptosomes prepared from the spinal cord of SOD1^G93A^ mice showed increased glutamate release both under basal conditions and after depolarizing stimuli, suggesting increased glutamate levels at the synapse biophase. Interestingly, this event was already detectable in 30-day-old SOD1^G93A^ mice in the early, pre-symptomatic stage of the disease [195,196]. Glutamate release was exocytotic and sustained by plastic changes in the release machinery protein expression, phosphorylation, and assembly.

As to astrocytes, the elevation of extracellular glutamate concentration was ascribed to the impaired glutamate clearance due to reduced expression of the astrocytic excitatory amino acid transporter 2 (EAAT2) [174,202,203]. In addition, astrocytes can actively release the excitatory amino acid. Astrocytes in culture derived from sporadic or SOD1^A4V^, but not *C9orf72* ALS patients, abnormally secrete glutamate in the culture medium [204]. Also, the GABA-induced glutamate release from spinal cord gliosomes, an in vitro functional preparation of the peri-synaptic astrocyte regions [205,206], was enhanced in SOD1^G93A^ mice, and this excessive release was very precocious, largely preceding the onset of the disease’s symptoms [207].

Thus, glutamate released by non-neuronal cells can represent an additional factor contributing to the increasing extracellular glutamate levels in ALS [208]. Of note, this increased glutamate availability can, in turn, dysregulate post-synaptic neuronal and neighboring glial cells. There are various mechanisms by which ALS astrocytes can act as producers of glutamate excitotoxicity, increasing the glutamate extracellular levels and simultaneously being the target of their released glutamate. The dual faces may be linked to each other. The excess of glutamate released by astrocytes in ALS will target heterologous cells in the brain and spinal cord, such as MNs, microglia and oligodendrocytes, as well as neighboring astrocytes contributing to further amplifying, in a vicious circle, their reactive phenotype and, consequently, also the toxic impact versus the neighboring neuronal and non-neuronal cells.

Globally, we believe that the emerging astrocyte roles should consider these cells to be “producers” and “targets” of glutamate excitotoxicity in ALS.

**Figure 2:**
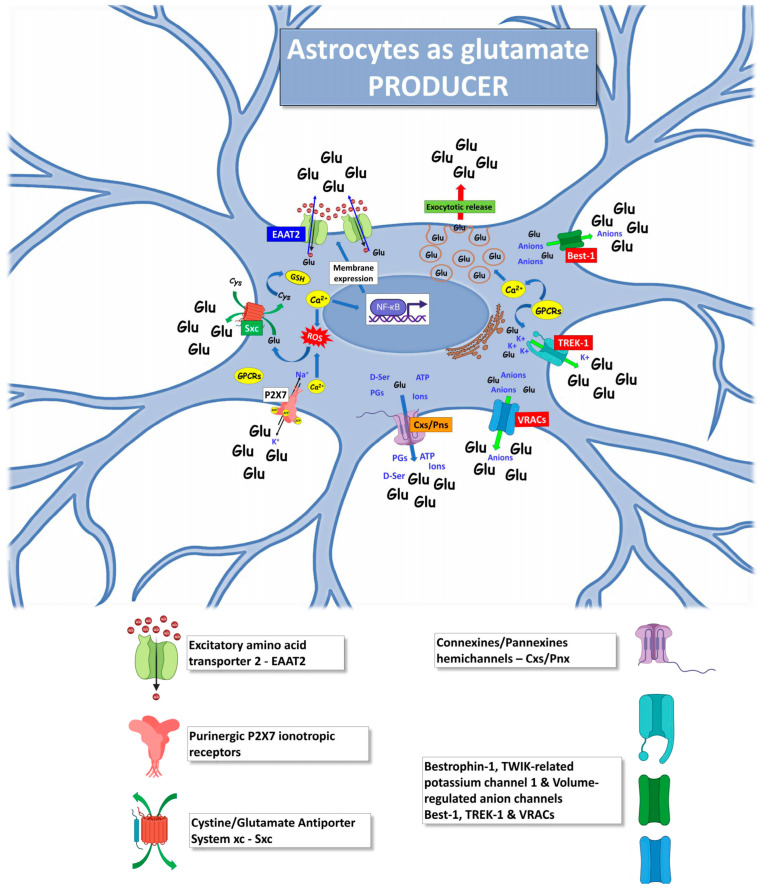
Mechanisms by which ALS astrocytes can act as producers of glutamate excitotoxicity, increasing the glutamate extracellular level. The excess of glutamate released by astrocytes will target heterologous brain and spinal cord cells, such as MNs, microglia, oligodendrocytes, and neighboring astrocytes. The figure was partly generated using BioRender.com (accessed on 25 July 2023) and Servier Medical Art, provided by Servier, licensed under a Creative Commons Attribution 3.0 unported license (https://creativecommons.org/licenses/by/3.0/ (accessed on 25 July 2023)).

**Figure 3:**
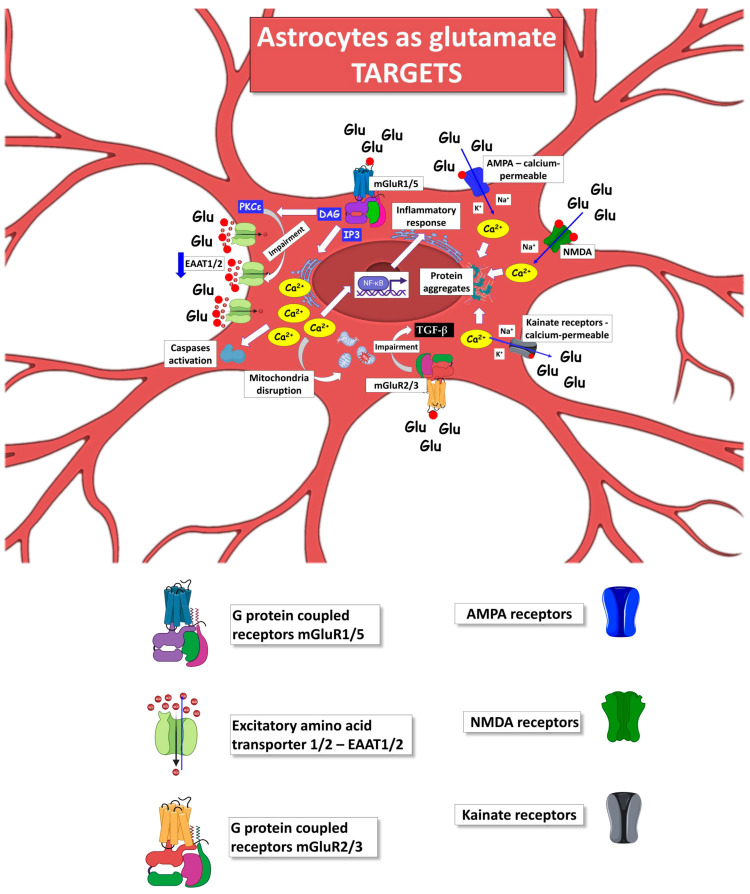
Mechanisms by which ALS astrocytes can act as the target of glutamate released by neighboring astrocytes, contributing to further amplifying their reactive phenotype and toxic impact versus neuronal and non-neuronal cells. The figure was partly generated using BioRender.com (accessed on 25 July 2023) and Servier Medical Art, provided by Servier, licensed under a Creative Commons Attribution 3.0 unported license (https://creativecommons.org/licenses/by/3.0/ (accessed on 25 July 2023)).

The mechanisms contributing to the role of astrocytes as producers of the excessive glutamate levels and targets of glutamate toxicity in ALS, described in the following chapters, are schematically represented in Figures 2 and 3.

## 4. Astrocytes as Producers of Excessive Glutamate in ALS

Astrocytes can actively contribute to defining the glutamate commitment in developing neuronal and glial damage during ALS progression. Several altered astrocyte mechanisms in ALS can impact the enhanced glutamate in the extracellular milieu and its action on MNs, astrocytes, microglia, oligodendrocytes, and other non-neural cells (Figure 2).

### 4.1. Excitatory Amino Acid Transporter 2

Abnormal glutamate availability represents one of the fundamental ALS-linked features. In 1995, EAAT2, expressed predominantly in astrocytes and responsible for about 90% of glutamate reuptake from the synapses, was found dysfunctional in the brain cortex and spinal cord astrocytes of ALS patients, causing impairment of the synapse glutamate clearance [174,209]. A further study revealed that EAAT2 functional alterations derived from aberrant truncated transcripts of the EAAT2 gene [210]. Many factors affect EAAT2 transcription, translation, and activity, such as oxidative stress, fatty acids, growth factors, or cytokines [211,212,213,214]. The reduced glutamate clearance leads to increased activation of the glutamate receptors of MNs with an abnormal influx of Ca^2+^, determining fatal changes in cell physiology and inducing ER stress, mitochondria overload, and cell death [215].

Recent research described the involvement of caspase 3 in regulating EAAT2 expression and function. Indeed, EAAT2 presents a caspase-3 consensus sequence cleaved by activated caspase-3 in vitro, thus determining the formation of truncated fragments of EAAT2 and their accumulation into the cells, inducing the release of neurotoxic substances from astrocytes in ALS. The generation of a double transgenic mouse carrying SOD1^G93A^ mutation and EAAT2D504N, a point mutation inhibiting the caspase 3 cleavage of EAAT2, produced slowed disease progression, characterized by delayed development of hind- and fore-limb muscle weakness and significant extension of the lifespan. However, the disease onset was not affected, suggesting other pathway involvement in regulating EAAT2 expression, and hindering the importance of EAAT2 impairment as a triggering cause of ALS [216].

Yin and colleagues described an interplay between the astrocyte elevated gene-1 (AEG-1) and EAAT2 expression in astrocytes. AEG-1 was up-regulated in primary cortical astrocyte cultures prepared from SOD1^G93A^ mice, leading to a decreased astrocyte membrane EAAT2 expression through NFκB activation. Silencing AEG-1 (siAEG-1) restores EAAT2 expression and glutamate uptake. Moreover, mutant-SOD1 neurons cultured with siAEG-1 astrocyte-conditioned medium improved viability. These results encourage the investigation of other pathways for AEG-1 in ALS and point to AEG-1 as a promising target for ALS pharmacological approaches [217]. Notably, valosin-containing protein (VCP) astrocytes, another gene causing ALS, showed a reduced glutamate uptake and enhanced reactive state, increasing proinflammatory signaling and becoming less supportive for neurons [218].

Overall, a significant reduction of EAAT2 in the motor cortex and spinal cord is one of the principal factors leading to glutamate excitotoxicity in ALS [219].

### 4.2. Exocytotic Glutamate Release

Ca^2+^-dependent astrocytic exocytosis is a long-lasting known occurrence in astrocytes [220,221]. The increase in intracellular Ca^2+^ concentration [Ca^2+^]_i_ can arise primarily by the mobilization of internal stores and may involve astrocyte-expressing receptor activation [222,223,224,225]. However, increased [Ca^2+^]_i_ involving external Ca^2+^ entry has also been reported after electrical or chemical depolarization of cultured astrocytes [226,227]. The KCl-induced depolarization evoked glutamate release has also been reported in adult astrocytes and peri-synaptic astrocyte processes (gliosomes) prepared from rat brain cortex [228], thus supporting the astrocytic excitability properties. Accordingly, astrocytes possess release machinery typical of neurons, such as the SNARE complex proteins and synaptotagmin-1, likely to produce regulated exocytosis [221,229,230]. The presence of vesicle-associated proteins, including the vesicular glutamate transporters, suggested the presence of glutamate-containing vesicles in the astrocyte active zone and cytoplasm, which were evidenced by electron microscopy and proteomic studies [205,206,231,232,233].

As the neuronal counterpart, whose exocytotic glutamate release has been found abnormal in SOD1^G93A^ mice after membrane depolarization or mGluR1/5 receptor activation [195,196,199,201,234], the exocytotic release from astrocytes is altered in ALS. Manfredi and colleagues analyzed Ca^2+^ homeostasis and exocytosis in SOD1^G93A^ mouse-derived astrocytes and found that ATP stimulation augmented [Ca^2+^]_i_ due to excessive Ca^2+^ release from endoplasmic reticulum (ER) stores and based on altered Ca^2+^ accumulation in the ER in SOD1^G93A^ astrocytes [235]. Astrocytic exocytosis inhibition in SOD1^G93A^ astrocytes preserved MNs from death in astrocyte-MN co-cultures and delayed the disease onset in SOD1^G93A^ mice without affecting disease progression, providing in vitro and in vivo evidence that astrocyte exocytosis contributes to ALS pathogenesis. However, no evidence was provided about glutamate exocytosis and its involvement in the effects described. Another study evidenced that immunoglobulins G (IgG) purified from patients with amyotrophic lateral sclerosis (ALS) enhanced the vesicle mobility in cultured rat astrocytes, which depended on the cytoplasmic Ca^2+^ homeostasis, with no apparent indirectly assessed release of their cargo most likely including glutamate [236].

Considering that the exocytotic mechanism represents one of the main processes for glutamate release by astrocytes, further studies are needed to gather compelling evidence that, besides altered neuronal release, astrocyte glutamate exocytosis is instrumental to ALS.

### 4.3. Purinergic P2X7 Receptors

The purinergic P2X7 receptor subtype (P2X7R) is a ligand-gated cation channel that provides another pathway for glutamate release from astrocytes [220]. ATP acts as a ligand of the receptor that, upon binding, facilitates the influx of the small cations, such as Ca^2+^ and Na^+^, and the efflux of K^+^. Instead, prolonged exposure to high ATP concentrations leads to the opening of a dilated membrane pore permeable to large molecules up to 900 Da, including glutamate [237,238]. The P2X7Rs are mainly localized on microglia cells but are also expressed on neurons, oligodendrocytes, and astrocytes [239,240]. Duan et al. provided the first evidence that these receptor channels could mediate the release of glutamate from astrocytes [241]. The release of glutamate through P2X7Rs is [Ca^2+^]_i_ independent, insensitive to voltage changes, and blocked by the P2X antagonists [220,242].

The main pathological function of P2X7Rs is during the inflammatory response and is characterized by increased extracellular ATP that activates the receptor and enables the maturation and release of cytokines, such as interleukin-18 (IL-18) and IL-1β [243]. In astrocytes, P2X7R stimulation potentiates the inflammatory cascade by enhancing the IL-1β-induced activation of NF-κB and the activator protein 1 (AP-1) transcription factors, increasing the production of NO, the monocyte chemoattractant protein-1 (MCP-1), and IL-8 chemokines [244,245].

The P2X7R has been found to be upregulated in microglia and astrocytes, resident in the spinal cord of ALS patients and SOD1^G93A^ animals, thus leading to the hypothesis that ATP signaling may trigger cytotoxic events in astroglial cells, resulting in proximal motor neuron damage [246,247]. Extensive preclinical studies identified the P2X7Rs as playing two roles in ALS: neuroprotective and neurodegenerative, depending on the disease stage, being predominantly neuroprotective in early disease stages while becoming gradually detrimental during ALS progression [248,249,250]. Moreover, the P2X7R pharmacological modulation displayed controversial gender-dependent effects in SOD1^G93A^ mice [251,252].

Gandelman and collaborators demonstrated that selective activation of the P2X7Rs in SOD1^G93A^ astrocytes led to motor neuron death, most likely due to releasing toxic factors, including glutamate. Indeed, the P2X7R is constitutively activated in SOD1^G93A^ astrocytes by increased extracellular ATP signaling [253], thus contributing to ATP-dependent exacerbation of the astrocyte neurotoxic phenotype [254]. Of note, supporting the role of ATP and the consequent P2X7R activation, the SOD1^G93A^ astrocyte-mediated MN death was significantly prevented by depleting ATP with apyrase or by blocking the P2X7R with the antagonist brilliant blue G (BBG) [254].

Considering the above evidence, astrocyte-expressed P2X7Rs play a role in ALS disease, contributing to oxidative stress, inflammatory signaling and glutamate-mediated neurotoxicity. Even though there are some controversies, the pharmacological or genetic inhibition of this receptor may facilitate the astrocyte switch to a more trophic phenotype toward neurons; however, further studies on ATP and P2X7R signaling in astrocytes are encouraged to understand better the potential contribution for the development of effective and cell-specific therapies in ALS [255].

### 4.4. Cystine/Glutamate Antiporter System xc

The cystine/glutamate antiporter system xc (Sxc) is a membrane heterodimer crucial to sustaining astroglial glutamate release in several CNS regions. It is an anionic amino acid antiporter that exports glutamate for cystine. Cystine is critical for glutathione synthesis and maintaining the cellular antioxidant pool [220]. Moreover, increased Sxc-mediated glutamate release was observed even before the EAAT2 reduction, thus contributing to the early glutamate toxicity during the disease initiation in the SOD1^G93A^ transgenic mouse model of ALS [256].

A recent study showed that the deletion of xCT (core protein of Sxc-) delayed the disease progression rate in the mutant SOD1^G37R^ ALS mouse model [257], thus confirming its essential role in driving the disease. Of note, the oxidant environment upregulates xCT, causing an increase in extracellular glutamate levels that, in turn, induce Ca^2+^-mediated excitotoxicity [258]. Since oxidant species are present during ALS progression, this exchanger could enhance glutamate excitotoxicity during the disease progression.

Although there is evidence for exact upregulation in ALS mouse models and postmortem spinal cords of ALS patients, there are a few discrepancies concerning the molecule location. In the genetic SOD1^G37R^ and SOD1^G85R^ mouse model, xCT levels in microglial cells were significantly upregulated in the spinal cord [257], whereas, in human ALS postmortem spinal cord tissues, xCT was specifically expressed and upregulated only in astrocytes [258]. This differential expression could be based on the differences of species between humans and mice.

Further analysis will better clarify the role of this mechanism in ALS astrocytes and its impact on the surrounding cellular environment.

### 4.5. Hemichannels

Connexins (Cx) and pannexins (Pn) are two membrane protein families forming hemichannels [259], creating connexons or gap junctions (GJs), allowing the exchange of molecules and ions as well as toxic substances, such as excitatory amino acids, with neighboring cells and promoting Ca^2+^ overload [260,261]. In the CNS, GJs are widely expressed in astrocytes, where they couple these cells to create a functional syncytium [262], also allowing glutamate release [263] and favoring the inflammatory response [264]. Astrocytes preferentially expressed Cx30 and Cx43 [265,266]. The selective activation of astrocyte Cx43favors ion diffusion and ATP, prostaglandin E2, D-serin, and gliotransmitter release [262,267,268,269,270]. The Cx-mediated glutamate release could, in turn, activate n-methyl-D-aspartate (NMDA) receptors, favoring Ca^2+^ oscillation in the same or nearby astrocytes [271] and regulating synaptic plasticity [272,273,274].

Accumulating evidence suggests a connection between ALS and Cxs [275,276]. Astrocytic GJ Cx43 was strongly dysregulated in the anterior horns of the spinal cords of mutant SOD1^G93A^ transgenic mice during disease progression and at the end stage, suggesting that the GJ disruption can aggravate MN death, contributing to glutamate excitotoxicity and ALS progression [277]. Keller and colleagues found an intimate connection between activated microglia and astrocytes via Cx43 at the end stage of ALS [278], thus supporting the idea that the altered Cx43 function affects microglia reactivity and the inflammatory response. As confirmation of this scenario, Cx43 overexpression was described in the SOD1^G93A^ mouse model as well as in post-mortem motor cortex, spinal cord, and cerebrospinal fluid derived from ALS patients; accordingly, neuroprotection through Cx43 blockers and Cx43 hemichannel blockers was shown to be beneficial [279]. Conversely, no noticeable Cx30 expression changes exist in SOD1^G93A^ mutant mice [277,279].

Although there is an apparent lack of Cx30 effect in slowing down ALS progression, this protein has an important role in regulating the inflammatory response. Cx30 deficiency increased microglia ramifications enlarged astrocytic processes, and reduced Cx43 expression [280,281]. Recent findings suggest, using a genetic approach that reduced expression of astroglial Cx30 in SOD1^G93A^ mice protects neurons at the early disease stage by attenuating astroglial inflammation [282] and, similarly, reducing Cx43 expression improved disease progression [283]. Further evidence confirming the importance of hemichannels in ALS has been proposed by Lehrer and collaborators, pointing out that insulin can block Cx31 and Cx43, inhibiting the release of toxic molecules, including glutamate, thus representing a potential pharmacological intervention for ALS [284].

Overall, further research on hemichannels expressed by astrocytes is needed to better individuate the toxic molecules that over-activate them and their possible negative effects on glutamate balance, astrocytes, or other neighboring cells during ALS progression.

### 4.6. Bestrophin-1, TWIK-Related Potassium Channel 1 and Volume-Regulated Anion Channels

Bestrophin-1 (Best-1) is an anionic channel activated by Ca^2+^. Its physiological activities include the release of molecules, such as glutamate, GABA, and chloride ions [285,286]. Best-1 is expressed in astrocytes and releases glutamate upon increased Ca^2+^ concentration, thus activating neuronal and non-neuronal NMDA receptors and, in turn, potentiating synaptic responses and modulating synaptic plasticity [287]. In specific pathological conditions, such as AD and PD, astrocytes undergo a phenotypic change, shifting from glutamate to GABA-releasing astrocytes through Best-1, thus affecting synaptic excitability and contributing to memory loss [288] and altered dopamine excitability [289]. No information about the Best-1 role in ALS is available to date. Due to the Best-1 role in other pathological conditions, studies on a possible contribution to ALS will help understand whether it excessively releases glutamate or shifts to GABA release, clarifying whether its activity needs to be blocked or enhanced.

The TWIK-related potassium channel 1 (TREK-1) is a type of K2P channel with a double-pore-domain background potassium channel [290]. In astrocytes, TREK-1 controls cell excitability by maintaining the membrane negative potential [291], and it mediates the passive potassium conductance and release of glutamate from astrocytes upon heterodimerization [292]. Given that these channels are in the soma and processes, instead of the perisynaptic domains, they limit their influence on post-synaptic receptors, namely metabotropic glutamate receptors (mGluRs) [293]. Several factors activating G protein-coupled receptors can cause fast astrocytic Ca^2+^-independent glutamate release from astrocytes through TREK-1; thus, this mechanism could enhance glutamate levels at the extracellular space, but further evidence is needed.

Similarly, volume-regulated anion channels (VRACs) release massive amounts of glutamate from swollen astrocytes, which could increase the extracellular amino acid level and overstimulate glutamate receptors in surrounding cells [294]. VRAC activity induces volume alteration in astrocytes, triggering NLRP3 activation and causing inflammation [295], and its activity is also upregulated in the presence of ROS [296]. Based on the role of these channels in contributing to glutamate excitotoxicity [297], it is reasonable to suppose their implication in ALS progression. However, to the best of our knowledge, no information is available.

### 4.7. Other Mechanisms Triggering Astrocytic Glutamate Excitotoxicity

A direct link between neuroinflammation and astrocyte-fostered glutamate excitotoxicity has been demonstrated [298]. In astroglia, the TNF-α interaction with its receptor TNFR1 induces a cascade of intracellular events leading to the generation of prostaglandin E2 that, in turn, activates intracellular Ca^2+^ elevation followed by glutamate exocytosis [223,299]. Moreover, TNF-α has a detrimental effect on astroglial glutamate uptake [300], downregulating EAAT2/GLT1 mRNA [301,302], thus inducing higher extracellular glutamate levels. TNF-α can also potentiate glutamate-mediated cytotoxicity by rapidly triggering the surface expression of Ca^2+^ permeable-AMPA and NMDA receptors while decreasing inhibitory GABA_A_ receptors on neurons. Thus, the net effect of TNF-α is to alter the balance of excitation and inhibition, resulting in a higher synaptic excitatory/inhibitory ratio [298].

Similarly, interleukin (IL)-1β and TNF-α dose-dependently inhibited astrocyte glutamate uptake by a mechanism involving nitric oxide, whereas interferon (IFN)-gamma alone stimulated this activity [303]. Moreover, an IL-1β, TNF-α, and IFN-γ cytokine mixture enhanced the calcium-dependent glutamate release from astrocytes induced by NO [304].

Inward rectifying Kir4.1 channels in astrocytes mediate spatial potassium (K^+^) buffering, a clearance mechanism for excessive extracellular K^+^, in tripartite synapses, and it is also essential for glutamate and water homeostasis in synapses [305]. Kir4.1 channels are functionally coupled to the glutamate transporters and the water transporter aquaporin-4 [306,307,308,309]. Although it is known that the astrocyte Kir4.1 channels regulate excitability and synaptic plasticity by controlling extracellular K^+^, glutamate clearance, and BDNF level in tripartite synapse, and are consequently involved in different brain diseases, less has been emerged in the ALS field. A reduction of Kir4.1 was observed in the brain and ventral spinal cord of asymptomatic animals [310] and altered Kir currents were observed in cultured SOD1^G96A^ astrocytes [311,312]. Thus, the dysregulation of the Kir4.1 channels in astrocytes might contribute to glutamate excitotoxicity and is considered a novel and promising therapeutic astrocyte link [313].

These mechanisms have not been fully evaluated in ALS; however, they are likely to have a role in disease progression where inflammation is a significant actor.

## 5. Astrocytes as Target of Glutamate Excitotoxicity in ALS

Glutamate toxicity is highly relevant in ALS since it may contribute to disease progression via multiple pathways, including a direct effect on the MNs and a modulation of the astrocytic reactive phenotype and their secretome, thus providing paracrine signals to neighboring cells. As described above, astrocytes can contribute to elevating glutamate excitotoxicity by several mechanisms and can sense glutamate becoming targets of their own released excitatory amino acid (Figure 3).

After being released, glutamate binds to several receptors, including the ionotropic N-methyl-D-aspartate (NMDA), α-amino-3-hydroxy-5-methy-4-isoxazolepropionic acid (AMPA), kainate receptors, and the metabotropic mGluRs, contributing to physiological and pathological events depending on the amplitude of the glutamate exposure. Excitotoxicity occurs when high levels of glutamate are present in the biophase, resulting in persistent activation of NMDA receptors, AMPA receptors, and voltage-gated calcium channels, generating a toxic influx of extracellular Ca^2+^.

Neuronal NMDA receptors mainly mediate glutamate-induced excitotoxicity; however, it is unclear whether astrocytes are involved in this phenomenon. To our knowledge, there is no evidence of a direct contribution of astrocytic NMDA receptors as glutamate targets affecting the astrocytes’ phenotype during ALS progression [314]. Also, no direct evidence exists for the other ionotropic glutamate receptors expressed by astrocytes in ALS [315].

Focusing on astrocytes as glutamate targets in ALS, it is fundamental to understand how excessive extracellular glutamate can change the astrocyte phenotype and reactivity, either by itself or with the contribution of other toxic molecules. It has been reported that extracellular glutamate can trigger astrocyte depolarization [316,317], which leads to the secretion of soluble factors, including glutamate itself [318].

Supporting the crucial role of astrocytes as a target of glutamate, Zuo and colleagues recently showed that excessive extracellular glutamate can activate astrocyte C3 expression and promote the release of pro-inflammatory factors, such as TNF-α and IL-1β [319]. However, the pathways responsible for this activation have not been elucidated. ALS can exacerbate this scenario due to the presence of high extracellular glutamate levels and mutant astrocytes, which are more reactive to toxic stimuli. Rossi and colleagues corroborated the above hypotheses, showing that in vitro exposure to glutamate resulted in focal degeneration of astrocytes cultured from the spinal cord of SOD1^G93A^ mice and not in WT-SOD1 mouse astrocytes [320]. The selective toxicity was triggered by activating specific astrocytic mGluRs [320], suggesting a possible mechanism for the glutamate-induced excitotoxicity in astrocytes. This evidence has also been recently confirmed by our research group [78].

In the paragraph below, we report the most relevant evidence of the involvement of astrocytic mGluR in ALS and the implications for potential therapeutic intervention.

### Metabotropic Glutamate Receptors

We know more about the altered expression and function of astrocytic mGluRs in ALS. In general, the stimulation of Group I, including mGluR_1_ and mGluR_5_, or Group II, including mGluR_2_ and mGluR3, expressed on reactive astrocytes leads to the release of harmful or protective substances, such as glutamate [321,322,323,324], BDNF [325], GDNF [326], and TGF-β [327,328]. Both Groups I and II mGluRs (mGluR1/5; mGluR2/3) are overexpressed in the astrocytes of ALS animal models and patients [199,329,330,331], thus being considered good pharmacological targets in ALS [332].

Of note, Group I mGluRs (mGluR_1_ and mGluR_5_) expressed on glutamatergic synapses localized in the spinal cord of SOD1^G93A^ ALS mice resulted in overexpression and enhanced sensitivity to the agonist present in the synaptic cleft; this phenomenon triggers increased activation of these receptors, mobilization of Ca^2+^ from the intracellular stores and further glutamate release [201]. These effects started at 90 days of life, an early symptomatic stage of the disease, and increased during the late phase. The over-expression of mGluR_1_ and mGluR_5_ at neuronal presynaptic sites paralleled the abnormal glutamate release described above in SOD1^G93A^ mice [195,196]. These receptors were also over-expressed at pre-symptomatic stages of the disease in the spinal cord tissue, embracing post-synaptic neuronal sites and non-neuronal cells, including astrocytes [199,201]. Accordingly, in vivo downregulation of mGluR_1_ or mGluR_5_ in the SOD1^G93A^ mouse model of ALS delayed the disease onset, slowed the disease progression, and prolonged survival. The increase in surviving MN numbers, together with a reduction of mitochondrial damage, down-regulation of oxidative markers, normalization of abnormal glutamate release, and decrease in astrocyte and microglia activation in the spinal cord, accompanied the slowing down of the disease progression [197,198,200]. Previous studies have revealed that mGluRs modulate excitatory synaptic transmission through various transduction pathways, i.e., by influencing the glutamate transporter expression in cultured astrocytes [333]. Astrocytes derived from an animal model of ALS carrying mutant SOD1 evidenced altered expressions and functions of mGluR_5_, which is involved in the activity and proliferation of astrocytes following damaging insults [333]. In accordance, primary astrocyte cultured from the brain cortex of SOD1^G93A^ rats showed a higher expression of mGluR_5_ than wild-type rats and dysregulation of the cross-talk between mGluR_5_ and EAAT2, leading to a lower number of Ca^2+^ oscillations and reduced glutamate clearance in the synapses [320,334,335]. The altered mGluR_5_ function derives from the downregulation of protein kinase C epsilon isoform (PKCε). Indeed, the restoration of PKCε in mutant SOD1^G93A^ astrocytes determined the normalization of Ca^2+^ oscillation and restoration of the dynamic mGluR_5_-dependent control of glutamate clearance by these cells. As a further proof-of-concept, PKCε silencing in wild-type astrocytes recapitulated the decreased Ca^2+^ oscillation observed in SOD1^G93A^ astrocytes [335].

The mGluRs strictly link excitotoxicity and neuroinflammation. Indeed, Berger and colleagues demonstrated that the expression of mGluR_3_ and mGluR_5_, the more expressed mGluRs in astrocytes, was affected by TNF-α and IL-1β in wild-type and mutant SOD1 astrocytes. Exposure to these proinflammatory cytokines down-regulated mGluR_5_ while up-regulating mGluR_3_, suggesting the presence of neuroprotective mechanisms [336]. Indeed, mGluR_5_ induced the synthesis of BDNF in astrocytes, which determines the sensitization of MNs to excitotoxic insults [325,337,338]. Conversely, mGluR_3_ promotes the release of TGF-β, thus protecting MNs from NMDA-induced excitotoxicity, and up-regulates the expression of glutamate transporters [326]. However, this regulatory mechanism could undergo alteration in ALS, at least at a late stage of the disease. Recently, it has been demonstrated that high extracellular glutamate levels increase the Lipocalin-2 (Lcn-2) concentration in the astrocyte cytoplasm by inducing a dose-dependent release of the protein via mGluR_3_ activation [339]. Since Lcn-2 is a key regulator of neuroinflammation, this mechanism could also be important in ALS.

In ALS, the elevated expression of mGluR_5_ makes astrocytes highly vulnerable to glutamate, causing aberrant and persistent elevations of intracellular Ca^2+^ concentrations [340] and inducing cells death [320]. On this basis, mGluR_5_ promises to be an excellent target to counteract disease progression. Genetic and pharmacological in vivo approaches have confirmed this hypothesis [198,200,320,337,341,342]. The beneficial effect of mGluR_5_ blockade was also confirmed in vitro since the genetic reduction or the pharmacological negative modulation of the receptor in late symptomatic SOD1^G93A^ mouse-derived spinal cord astrocytes ameliorates their reactive, inflammatory, bioenergetic, and neurotoxic phenotype [78]. These data support glutamate as an enhancer of the astrocyte reactive, proinflammatory, and neurotoxic phenotype in ALS in response to extracellular glutamate via mGluR_5_.

Other mechanisms could exacerbate the aspects described above. For instance, even if not confirmed in ALS, mGluR_5_ up-regulation contributed selectively to the apoptosis of astrocytes via the activation of phospholipase C and the release of calcium from intracellular stores as well as via the association with Homer proteins [343]. Further studies are needed to clarify better the molecular factors linking mGluR activation and the resulting reactive and toxic astrocytic phenotype, thus highlighting new potential targets in ALS. However, based on the literature, mGluRs, particularly mGluR_3_ and mGluR_5_, are undoubtedly primary mediators of the direct effects of glutamate excitotoxicity on astrocytes in ALS. This is why their pharmacological or genetic modulation is still considered a promising approach to disease treatment.

## 6. Concluding Remarks

Research on ALS has progressively shown a peculiar turning point in the approach to the disease. Attempts to gain a deeper understanding of the molecular mechanisms and cellular processes underlying the disease redirect the focus to MNs and have focused the attention of scientists on glial cells and their impact on MN degeneration. It soon became evident that all glial cells (astrocytes, microglia, and oligodendrocytes) are prone to the disease and can foster the progression of ALS.

Astrocytes substantially affect MN wellbeing and survival since they can damage MNs by non-cellular autonomous processes. In ALS, astrocytes undergo molecular and cellular changes that are harmful to MNs, leading to irreversible neurodegenerative processes. The mechanisms of a non-neuronal cell-driven MN death are closely related to the pathophysiological changes in ALS during disease progression, the characteristics of which strongly suggest that they differ from the autonomous cellular pathways. In this context, astrocytes play a fundamental role, intervening directly or indirectly in the modulation of almost all the mechanisms proposed at the basis of MN death.

This review analyzed how astrocytes regulate excitotoxicity, inflammation, oxidative stress, mitochondria function, and energy metabolism in ALS and how glutamate contributes to these pathological astrocytic mechanisms. Importantly, we highlighted that astrocytes may participate in these processes by enhancing glutamate secretion and its extracellular levels (producers) and abnormally responding to the augmented glutamate concentration (targets). Indeed, the glutamate excess produced by astrocytes can affect heterologous cells, including astrocytes that will become, in this way, the target of their released glutamate, exacerbating, or even triggering, abnormal astrocyte activation during ALS progression. This aspect, although impacting the disease course, has not been widely investigated in ALS; therefore, it deserves future studies that are able to unveil alternative therapeutic strategies that focus on the selective interception of pro-death signals in a cell-type-specific way.
